# Bone Regeneration Using a Mixture of Silicon-Substituted Coral HA and β-TCP in a Rat Calvarial Bone Defect Model

**DOI:** 10.3390/ma9020097

**Published:** 2016-02-06

**Authors:** Jiyeon Roh, Ji-Youn Kim, Young-Muk Choi, Seong-Min Ha, Kyoung-Nam Kim, Kwang-Mahn Kim

**Affiliations:** 1 BK21 PLUS Project, Yonsei University College of Dentistry, Seoul 03722, Korea; hindhorn@gmail.com (J.R.); kimkn@yuhs.ac (K.-N.K.); 2 Department and Research Institute of Dental Biomaterials and Bioengineering, Yonsei University College of Dentistry, Seoul 03722, Korea; 3 Department of Dental Hygiene, College of Health Science, Gachon University, Incheon 21936, Korea; hoho6434@gmail.com; 4 Meta-Biomed Co., Ltd. Institute, 270 Osongsaengmyeong1-ro, Osong-eup, Heungdeok-gu, Cheongju-si, Chungbuk 28161, Korea; dudanr@metabiogw.bizmeka.com (Y.-M.C.); haseongmin@metabiogw.bizmeka.com (S.-M.H.)

**Keywords:** silicon-substituted coral hydroxyapatite, beta-tricalcium phosphate, bone graft material, bone regeneration, rat calvarial bone defect model

## Abstract

The demand of bone graft materials has been increasing. Among various origins of bone graft materials, natural coral composed of up to 99% calcium carbonate was chosen and converted into hydroxyapatite (HA); silicon was then substituted into the HA. Then, the Si-HA was mixed with β-tricalcium phosphate (TCP) in the ratios 100:0 (S100T0), 70:30 (S70T30), 60:40 (S60T40), and 50:50 (S50T50). The materials were implanted for four and eight weeks in a rat calvarial bone defect model (8 mm). The MBCP^TM^ (HA:β-TCP = 60:40, Biomatalante, Vigneux de Bretagne, France) was used as a control. After euthanasia, the bone tissue was analyzed by making histological slides. From the results, S60T40 showed the fastest bone regeneration in four weeks (*p* < 0.05). In addition, S60T40, S50T50, and MBCP^TM^ showed significant new bone formation in eight weeks (*p* < 0.05). In conclusion, Si-HA/TCP showed potential as a bone graft material.

## 1. Introduction

Bone graft materials are in high demand for the replacement of bone tissues due to accidents, diseases, or other implantation needs. Various bone graft materials have been introduced, and an autograft or allograft was regarded as the gold standard. However, there were many limitations, such as bone quantity, risks of immune responses, and disease transmission [1]. Therefore, chemically-synthesized biomaterials, such as hydroxyapatite (HA) and β-tricalcium phosphate (TCP), have become preferred bone graft materials of choice [2]. HA has achieved significant application in a range of medical and dental applications. However, due to its slow rate of degradation, components with faster degradation rates were added; among them, β-TCP has been used as one of the main components of bone graft materials [3]. In many products, the degradation rate is controlled by changing the ratio of HA to β-TCP, and so many products have a HA:β-TCP ratio of 60:40 or 70:30. 

HA can be obtained from biological and natural sources, such as coral and seashells [4]. In this study, we used raw sea coral to synthesize HA. Coral is pure calcium carbonate (with up to 99%) and little amino acid and oligo elements. It is easily obtained in large quantities from nature and the risk of infection is lower than with an allograft [5]. The coral genus Goniopora was used as raw material here. It has a porous structure and the pores are interconnected similar to bone. We have synthesized this into HA in previous studies [6,7].

Silicon is one of the most abundant elements on earth and it is an important element in the body. It has been detected in cartilage and other connective tissues [8]. Si is known as a key element in the early stages of the biomineralization process and it was reported as an HA inducer [9,10,11,12,13]. From the previous studies, we choose the amount of silicon and we substituted approximately 1 wt % of silicon in coral HA [6]. 

Thus, using a synthesized Si-HA mixed with various ratio of β-TCP, we evaluate its bioactivity in an animal model. The objective of this study was to evaluate osteoconductivity of new bone graft material groups in a bone-defect model. 

## 2. Results

### 2.1. Radiological Findings after Four Weeks and Eight Weeks

**Figure 1:**
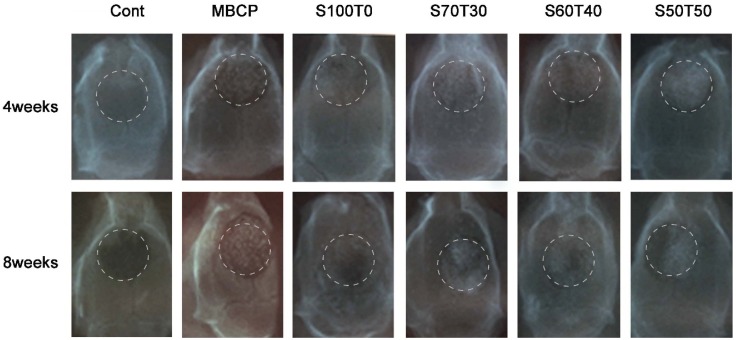
X-ray pictures (four weeks and eight weeks); Cont is the blank control group.

The X-ray results are shown in Figure 1. The granule materials were placed in the defect area and it was marked with circle. In addition, the surgical sites of all groups, except the control, were radiopaque even after eight weeks.

### 2.2. Histological Findings at Four Weeks and Eight Weeks

**Figure 2:**
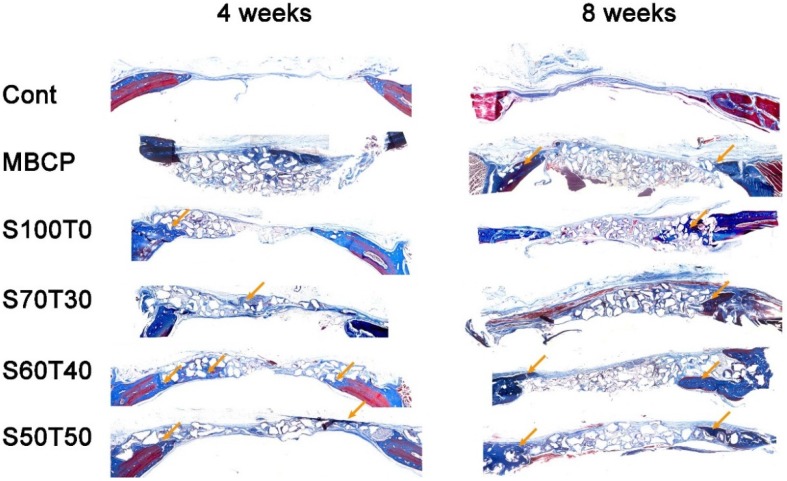
Histological slides with Masson’s Trichrome staining (four weeks and eight weeks).

The histological slides after four and eight weeks are shown in Figure 2. The original and new bone was stained with blue and an osteoid was in red (arrows). There were only connective tissues in blank control groups at four and eight weeks. Although all groups showed that the materials remained unabsorbed, the number particles had decreased after eight weeks compared with four weeks. In eight weeks, there were embedded bone graft particles in new bones (S70T30 and S60T40).

### 2.3. Quantified Results of Histological Slides (Four Weeks and Eight Weeks)

**Figure 3:**
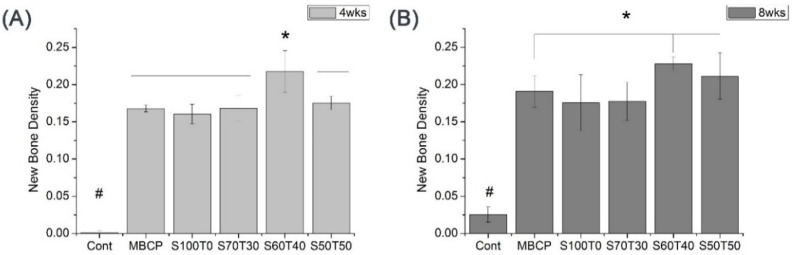
Quantitative results of new bone formation: (**A**) four weeks; and (**B**) eight weeks.*, # means significant differences (*p* < 0.05).

The new bone density is quantified in Figure 3. In four weeks (Figure 3A), there was no bone formation in the blank control (Cont). The S60T40 was significantly higher than others (*p* < 0.05), however, there were no significant differences among the MBCP, S100T0, S70T30, and S50T50. In eight weeks (Figure 3B), there was new bone formation in the blank control (Cont). The S60T40, S50T50, and MBCP^TM^ were significantly higher than S100T0 and S70T30 (*p* < 0.05). 

## 3. Discussion

Hydroxyapatite (HA) has achieved significant application as a bone graft material in medical and dental applications. HA, generally, are based on a chemical reaction between calcium and phosphorous salts and it can also be obtained from biological sources such as coral and sea shells. One of the advantages of HA derived from natural sources is that it already contains the minor elements, which can have beneficial effects on HA properties [4]. This study was designed to evaluate new bone graft materials containing naturally-derived Si-HA and β-TCP mixture in a bone defect model. In the previous study, the natural coral was used as scaffold with its interconnected pores throughout its structure because it resembles the microstructure of trabecular bones. In addition, the coral met the requirements of bone graft materials in quantity and purity. It is made up of 99% calcium carbonate and, through the thermal process, the CaCO_3_ transformed to HA and any amino acids, oligo elements, and other impurities were eliminated [7]. We used the coral in granule form, and during the process the macroscale structure was collapsed. However, the micro and interconnected structures were reserved. 

In bone remodeling various factors such as, mineral elements, cells, and extracellular membranes, and many growth factors are required. In addition, the micro-environment has been an important factor to start tissue regeneration at the early stage of bone repair. In this study, we focused on silicon, which has been known as an accelerator in bone mineralization. During the process of converting coral into HA, we substituted Si ions into the HA structure. In a previous study, Carlisle *et al.* reported that Si played an integral role during the mineralization process and it has been reported as a fundamental element in collagen [9]. Porter *et al.* had conducted an implantation test, and after six and 12 weeks they reported that although both pure HA and Si-HA showed good results, organized collagen fibrils were found in the Si-HA implants after six weeks. However, these structures were found only after 12 weeks in pure HA implants [14]. 

The HA:β-TCP ratio of 60:40 has been designated the gold standard due to its ideal dissolution rate. In the previous study of HA and TCP graft at 60:40 ratio, they reported that 26%–40% of new bone was formed in bone defect model, and 27% of material remained after 4–9 months [15]. Although little is known about the effect of Si on cells, there was histological evidence of new bone formation in the biological response to an implant [13] and also we could confirmed in the S60T40 group after four weeks (Figure 2 and Figure 3A), and we suggested that this may be osteoconductive. 

From the Si-substituted HA, we expected the released Si to affect new bone formation and, indeed, the S60T40 (Si-HA/β-TCP) showed significantly higher bone formation than MBCP^TM^ (HA/β-TCP) in four weeks. The dissolution rate of HA *in vivo* was influenced by how the Si incorporated into the HA lattice [14]. The weight percentage of Si in HA may increase the solubility of the material [16,17]. Poter *et al.* also reported that Si increases the dissolution rate of HA. Dissolution was observed to be the highest with 1.5 wt % Si-HA, followed by 0.8 wt % Si-HA, and pure HA. It was found to be particularly prevalent at grain boundaries and triple-junctions and these observations may help to explain the mechanism of dissolution in Si-HA compared to pure HA [14]. From the result, if the *in vivo* bioactivity is related to this dissolution-reprecipitation mechanism, the results of this study, which had having used materials of approximately 1 wt % HA were as expected [6]. In this study, we compared the new bone density between S60T40 (Si-HA:β-TCP = 6:4) and MBCP^TM^ (HA:β-TCP = 6:4), S60T40 showed significantly higher values in four weeks.

The critical-size defect in rat calvaria has been regarded as the most desirable model because its poor blood supply and membranous structure of the bone precludes any natural healing. The defect should be the smallest diameter intraosseous wound possible without natural bone healing. We made an 8 mm defect, and the blank control confirmed that there was no bone regeneration at the defect site [18,19,20,21,22].

The histological slides provide qualitative evidence that the defect areas were not filled completely. In Figure 2 and Figure 3A,B, the granules still remained in the area from 4 to 8 weeks. Similar results had been reported after 10 weeks in a previous study [23]. In addition, the speed of new bone formation had slowed by eight weeks. It seems that during the first four weeks, the materials can reach bodily fluids. However, after collagen and new bone formed, the materials were incorporated into the new bone, and the possibility of contact with bodily fluids had decreased. After four weeks, there was little bone formation and the materials maintained their original morphology. On the other hand, although the materials still remained in the defect site, more particles had dissolved by eight weeks. According to the blank control, the bone healing started from the margins of the defect, and connective fibrous tissues came into the defect before bone formation. However, in the eight week experiment groups such as S60T40 and S70T30, there were embedded bone graft particles in new bone at the margin of the defect.

From the X-ray results, quantitative densitometric analysis for new bone formation could not be analyzed properly due to the material’s radiopaque nature, so we could not check noticeable bone regeneration results. We just confirmed that the materials stayed in the defect hole and the bone graft materials had tightened under the periosteal membrane even after eight weeks.

In conclusion, we successfully made Si-substituted coral HA from natural coral and with various ratios to β-TCP. We confirmed S60T40 implants had significantly more new bone formation in four weeks and eight weeks. It was clear that Si plays an important role in bone mineralization. It is, therefore, of future interest to investigate the other effects of S60T40 on the process of osteogenesis.

## 4. Experimental Section

### 4.1. Preparation of Si-Substituted Coral HA and β-TCP Mixture

#### 4.1.1. Preparation and Characterization of Si-Substituted Coral HA

The natural Goniopora coral (CaCO_3_, sGulf of Mannar, Indonesia) with interconnected pore sizes of 300–500 μm was purchased. Briefly, the purchased corals were immersed in NaOCl (12%–13%) for 24 h and then sonicated in distilled water to remove organic matter and impurities. The coral was cut into blocks and placed in a hydrothermal reactor with silicon tetra acetate-acetone solution (Sigma-Aldrich, St. Louis, MO, USA). After reacting for 24 h at 200 °C, the CaCO_3_ was converted into HA. Then, it was immersed in tetraortho silicate solution (TEOS, Sigma-Aldrich, St. Louis, MO, USA) and reacted at 60 °C for 48 h to synthesize silicon-substituted HA. Approximately 1 wt % of silicon was substituted into the HA structure. Si-HA was synthesized and characterized as described in reference [6].

#### 4.1.2. Synthesis of β-TCP

We synthesized β-tricalcium phosphate (β-TCP) in this study. Briefly, calcium nitrate tetrahydrate solution (Ca(NO_3_)_2_⋅4H_2_O, Junsei, Chuo-ku, Japan) and potassium dihydrogen phosphate solution (KH_2_PO_4_, Junsei, Chuo-ku, Japan) were mixed under stirring and the mixed solution was filtered. The filtered powder was dried and mixed with dispersant to make slurry. The slurry was absorbed into a polyurethane sponge (PU sponge, 80 ppi) and dried for 24 h. The dried sponge was sintered at 1100 °C until the PU sponge burnt away. Finally, the sintered β-TCP block was milled into granules, sized 100–600 μm.

#### 4.1.3. Si-Substituted Coral HA and β-TCP Mixture

To make the final composition of experimental bone graft materials, the Si-substituted coral HA and TCP granules were mixed homogeneously at ratios of 100:0, 70:30, 60:40, and 50:50. 

### 4.2. Materials for Animal Experiments

To evaluate bone healing ability, we conducted animal experiments with each material in Table 1. The MBCP^TM^ (Biomatlante, Vigneux de Bretagne, France) was used as the control product. We used various Si-substituted coral HA and TCP mixtures as experimental groups, which were described in Section 4.1.

**Table 1:** Experimental groups used in this study.

Groups	Label	Pure HA	Si-Substituted Coral HA	β-TCP
Blank control group	Cont	-	-	-
Control product group	MBCP	60	-	40
Experimental groups	S100T0	-	100	0
	S70T30	-	70	30
	S60T40	-	60	40
	S50T50	-	50	50

### 4.3. In Vivo Determination of Bone Regeneration of Bone Defect Model

**Figure 4:**
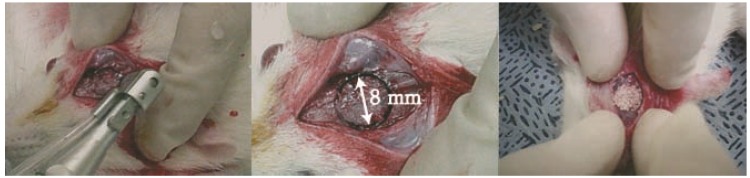
The surgical process (8 mm defect criteria).

All animal procedures were approved by the Institutional Animal Care and Use Committee (IACUC), Yonsei University Biomedical research institute (No. 06-236). Sixty specific-pathogen-free (SPF) Sprague-Dawley Rats weighing between 250 and 300 g underwent operation. Before surgery, the rats were monitored during seven days of stabilization. The rats were divided into two groups (four weeks and eight weeks) with six animals in each group. For the blank control and product control, we used three rats each. Briefly, the instruments were sterilized by autoclave and the materials were prepared. General anesthesia was induced by intramuscular injection (IM) with an anesthesia cocktail solution (0.1 mL/10 g) of a mixture of Zoletil (Zoletil 50 Vibrac, Carros cedex, France) and Xylazine (Rompun, Bayer Korea, Seoul, Korea). The calvaria fur was clipped and surfaces were sterilized by iodine. Local anesthesia of 2% lidocaine and 1:100,000 epinephrine (Lignospan Standard, Septodont, New Castle, DE, USA) was injected. The incision was drawn sagittal through the skin and the periosteum at the midline of the calvaria. Then, the flap was reflected and an 8 mm bone defect was created by a trephine bur under sterile saline irrigation. After puncturing the calvaria bone, implant materials were placed carefully in the hole. Then, the defect was covered by the periosteum and stitched with absorbed silk (Vicryl 4-0, Ethicon, Norderstedt, Germany) and the skin was stitched with black silk. The rats were euthanized after four or eight weeks. Under anesthesia, the perfusion fixation was performed with 4% paraformaldehyde. The calvaria were removed and we took a periapical X-rays. The fixed tissues were subjected to X-ray imaging at 65 ± 5 kVp for 0.1 s. In this case, a distance between an X-ray tube and the tissues was set to 10 cm. The photographed X-ray film was developed and cone defect regions were then observed on the developed X-rays image. For the histological slides, the tissues were decalcified with 0.5 M Ethylenediaminetetraacetic acid (EDTA, Welgene, Korea) for three months and the bone tissues were made into histological slides (7 μm) and stained with Massons’ Trichrome. The surgical procedures are shown in Figure 4.

### 4.4. Histological Evaluation

To evaluate new bone formation, all histological slides were photographed at ×400 magnification. First, the defect area (A_D_) in the histological slide was outlined using Photoshop (Adobe Photoshop 7.0, San Jose, CA, USA) and calculated. The areas of new bone were selected and calculated (A_N_). The new bone density was calculated as A_N_/A_D_ [24].

### 4.5. Statistics

The histological results were processed using the SPSS Statistics 20 (SPSS Inc., Chicago, IL, USA). The one-way Analysis of variance for variance analysis with Tukey's multiple comparison was performed and the *p* values under 0.05 were considered statistically significant.

## 5. Conclusions

In this study, the Si-substituted coral HA and β-TCP mixture in the ratio of 60:40 showed superior osteogenic properties to pure HA in the rat calvarial bone defect model.
